# Diagnostic ultrasound estimates of muscle mass and muscle quality discriminate between women with and without sarcopenia

**DOI:** 10.3389/fphys.2015.00302

**Published:** 2015-10-29

**Authors:** Catheeja Ismail, Johannah Zabal, Haniel J. Hernandez, Paula Woletz, Heather Manning, Carla Teixeira, Loretta DiPietro, Marc R. Blackman, Michael O. Harris-Love

**Affiliations:** ^1^Muscle Morphology, Mechanics and Performance Laboratory, Clinical Research Center - Human Performance Research Unit, Veterans Affairs Medical CenterWashington, DC, USA; ^2^Department of Medicine, School of Medicine and Health Sciences, The George Washington UniversityWashington, DC, USA; ^3^Department of Physical Therapy and Health Care Sciences, School of Medicine and Health Sciences, The George Washington UniversityWashington, DC, USA; ^4^Department of Health Sciences, Malek School of Health Professions, Marymount UniversityArlington, VA, USA; ^5^Physical Medicine and Rehabilitation Service, Veterans Affairs Medical CenterWashington, DC, USA; ^6^Department of Exercise and Nutritional Sciences, Milken Institute School of Public Health, The George Washington UniversityWashington, DC, USA; ^7^The School of Kinesiology and Health Studies, Queen's UniversityKingston, ON, Canada; ^8^Departments of Biochemistry and Molecular Medicine, School of Medicine and Health Sciences, The George Washington UniversityWashington, DC, USA; ^9^Departments of Medicine and Rehabilitation Medicine, Georgetown University School of MedicineWashington, DC, USA; ^10^The Johns Hopkins University School of Medicine, Johns Hopkins UniversityBaltimore, MD, USA; ^11^Research Service, Veterans Affairs Medical CenterWashington, DC, USA

**Keywords:** sarcopenia, diagnostic ultrasound, geriatric assessment, body composition, dual-energy X-ray absorptiometry, muscle performance, muscle strength, myosteatosis

## Abstract

**Introduction:** Age-related changes in muscle mass and muscle tissue composition contribute to diminished strength in older adults. The objectives of this study are to examine if an assessment method using mobile diagnostic ultrasound augments well-known determinants of lean body mass (LBM) to aid sarcopenia staging, and if a sonographic measure of muscle quality is associated with muscle performance.

**Methods:** Twenty community-dwelling female subjects participated in the study (age = 43.4 ± 20.9 years; BMI: 23.8, interquartile range: 8.5). Dual energy X-ray absorptiometry (DXA) and diagnostic ultrasound morphometry were used to estimate LBM. Muscle tissue quality was estimated via the echogenicity using grayscale histogram analysis. Peak force was measured with grip dynamometry and scaled for body size. Bivariate and multiple regression analyses were used to determine the association of the predictor variables with appendicular lean mass (aLM/ht^2^), and examine the relationship between scaled peak force values and muscle echogenicity. The sarcopenia LBM cut point value of 6.75 kg/m^2^ determined participant assignment into the Normal LBM and Low LBM subgroups.

**Results:** The selected LBM predictor variables were body mass index (BMI), ultrasound morphometry, and age. Although BMI exhibited a significant positive relationship with aLM/ht^2^ (adj. *R*^2^ = 0.61, *p* < 0.001), the strength of association improved with the addition of ultrasound morphometry and age as predictor variables (adj. *R*^2^ = 0.85, *p* < 0.001). Scaled peak force was associated with age and echogenicity (adj. *R*^2^ = 0.53, *p* < 0.001), but not LBM. The Low LBM subgroup of women (*n* = 10) had higher scaled peak force, lower BMI, and lower echogenicity values in comparison to the Normal LBM subgroup (*n* = 10; *p* < 0.05).

**Conclusions:** Diagnostic ultrasound morphometry values are associated with LBM, and improve the BMI predictive model for aLM/ht^2^ in women. In addition, ultrasound proxy measures of muscle quality are more strongly associated with strength than muscle mass within the study sample.

## Introduction

Age-related declines in strength typically begin during the 4th decade of life, and range from 0.6 to 1.3% per year in people over 65 years of age (Hughes et al., 2001; Kamel, 2003; Frontera et al., 2008). Sarcopenia, an age-related loss of muscle mass that contributes to diminished muscle power and independent mobility, has been noted as a significant cause of morbidity in older adults (Janssen et al., 2004; Mitchell et al., 2012). The pathogenesis of sarcopenia is multifactorial and likely involves inflammatory, endocrine, neurological, and behavioral contributors. Importantly, the strength changes in older adults are often accompanied by myosteatosis, an increase in intramuscular adipose and connective tissue, along with the concomitant decrease in skeletal muscle cross-sectional area (Vandervoort, 2002; Kamel, 2003). These changes in *muscle quality* (e.g., muscle tissue composition, metabolic efficiency, or altered mechanics) may negatively impact functional performance in both women and men. Moreover, increased myosteatosis has been shown to be associated with decreased bone mineral density and lean body mass (LBM) in older women (Song et al., 2004).

Diminished LBM, muscle tissue composition, and muscle performance, are significant contributors to geriatric syndromes such as sarcopenia and frailty, and merit focused attention regarding standardized assessment and rehabilitation intervention strategies. Despite the substantial clinical and financial burden attributed to sarcopenia, it remains an under-diagnosed condition that is rarely subject to a systematic screening process for older adults (Fielding et al., 2011). The most commonly used LBM criterion for sarcopenia staging is appendicular lean mass (aLM, also expressed as aLM/ht^2^), as measured by dual energy X-ray absorptiometry (DXA) (Cruz-Jentoft and Morley, 2012; Malmstrom et al., 2013). However, due to space requirements for DXA, initial equipment costs, body size constraints, and general barriers related to specialized LBM assessment software and examiner training, DXA assessment of aLM is not an ideal measure for large scale sarcopenia clinical trials, bedside assessment, or community health screening efforts. Individual attributes such as age and sex are meaningful determinants of LBM, and alternative anthropometric methods have been used to estimate LBM (Harris-Love et al., 2014a). In addition, BMI has been shown to explain a significant proportion of the variance in LBM values (Iannuzzi-Sucich et al., 2002). However, these alternative estimates of LBM have limited utility as proxy measures, and the standard DXA examination does not provide information concerning muscle quality.

The use of diagnostic ultrasound for body composition assessment has been explored in concurrent validity studies involving DXA, hydrostatic weighing, and computed tomography (CT) imaging (Pineau et al., 2007; Utter and Hager, 2008). Also, sonographic characteristics of skeletal muscle have been associated with density values from magnetic resonance imaging (MRI) (Abe et al., 1994) and hydrodensitometry (Sanada et al., 2006) in Japanese adults. Unlike DXA, but similar to magnetic resonance and CT imaging, diagnostic ultrasound may be used to assess muscle quality via tissue characteristics. Muscle quality may be assessed via diagnostic ultrasound due to the hyperechoic nature of the non-contractile tissue associated with myosteatosis (Sipilä and Suominen, 1993). The use of diagnostic ultrasound for muscle tissue characterization has also been successful in the detection of various disorders such as Duchenne muscular dystrophy (Cady and Gardener, 1983; Berger et al., 1987; Schapira et al., 1987; Hughes et al., 2007). Moreover, the analysis of muscle tissue acquired via biopsy suggests that echogenicity is more strongly associated with intramuscular adipose tissue rather than fibrosis (Reimers et al., 1993). Consequently, diagnostic ultrasound may be a practical alternative approach to the assessment of both muscle mass and muscle quality. While there is some evidence to support the use of diagnostic ultrasound to estimate LBM (Sanada et al., 2006; Pineau et al., 2007; Utter and Hager, 2008), this method of body composition analysis is not widely used for sarcopenia screening and staging. Currently, diagnostic ultrasound is not identified as an accepted method to determine LBM by the major international sarcopenia consensus groups (Cruz-Jentoft et al., 2010; Lee et al., 2013; Dam et al., 2014). Therefore, the objectives of this pilot study are to examine if a rapid assessment method via mobile diagnostic ultrasound augments well-known determinants of LBM to aid sarcopenia staging, and if a sonographic measure of muscle quality is associated with muscle performance.

## Materials and methods

### Participants

Twenty community-dwelling women were enrolled for participation in the study at the George Washington University (GW) Exercise Physiology Lab in Washington, DC. The study was approved by the GW Office of Human Research Institutional Review Board, and registered with Clinicaltrials.gov (NCT00303446). Signed informed consent was obtained from all study participants prior to data collection. Inclusion criteria for study enrolment included being an ambulatory female adult between the ages of 18 and 75 years of age. This sample of convenience was stratified to include an equal number of people above and below the age of 55. Federal agencies have identified the age range of 55–65 as a benchmark period to observe the emergence of age-related health problems within U.S. populations (Schoenborn and Heyman, 2009). Absolute contraindications included pregnancy, medical conditions that result in edema, and musculoskeletal or neurological disorders that are associated with muscle atrophy. Relative contraindications were body size dimensions that would preclude appropriate use of the DXA scanner. Participant demographics are summarized in Table 1.

**Table 1:** **Participant characteristics**.

**Subject characteristics**	**All subjects (*N* = 20)**	**Normal LBM (*N* = 10)**	**Low LBM (*N* = 10)**	**Sig**.
Age (years)	43.4 ± 20.9	47.9 ± 21.3	39.0 ± 20.4	0.351
BMI^†^	23.8 (8.5)	28.8 (9.4)	21.5 (3.1)	0.001
aLM/ht^2^ (kg/m^2^)	6.96 ± 1.22	7.92 ± 0.88	6.00 ± 0.55	< 0.001
Grip strength (kg_*F*_/kg_*BW*_)	0.392 ± 0.089	0.345 ± 0.095	0.438 ± 0.054	0.017
Muscle thickness (cm)				
Trapezius	1.20 ± 0.19	1.27 ± 0.20	1.12 ± 0.15	0.076
Brachioradialis	1.95 ± 0.35	2.06 ± 0.40	1.84 ± 0.27	0.170
Deltoid	2.29 ± 0.53	2.54 ± 0.48	2.04 ± 0.45	0.031
Pectoralis	0.78 ± 0.23	0.85 ± 0.28	0.70 ± 0.15	0.163
major				
Rectus femoris	2.17 ± 0.54	2.34 ± 0.57	2.00 ± 0.48	0.157
Total muscle thickness (cm)	8.39 ± 1.18	9.07 ± 1.12	7.70 ± 0.81	0.006
Echogenicity^†^^‡^	47.50 (23.00)	58.50 (21.00)	38.00 (17.00)	0.003
Racial/ethnic group				
Caucasian	9 (45.0%)	3 (30.0%)	6 (60.0%)	–
African	4 (20.0%)	4 (40.0%)	0 (0.0%)	–
American				
Hispanic	2 (10.0%)	2 (20.0%)	0 (0.0%)	–
Asian	5 (25.0%)	1 (10.0%)	4 (40.0%)	–
HAQ	0.45 ± 1.10	0.50 ± 0.97	0.40 ± 1.27	0.605
Audit-C^†^	2.0 (2.0)	2.0 (2.0)	2.0 (2.0)	0.586

*LBM, lean body mass; sig, significant; BMI, body mass index; aLM/ht^2^, appendicular lean mass scaled to height; F, force; BW, body weight; HAQ, The Health Assessment Questionnaire; Audit-C, The Alcohol Use Disorders Identification Test*.

*Data expressed as means (± standard deviation); statistically significant differences between the Normal LBM subgroup and the Low LBM subgroup were determined using the independent t-test (p < 0.05)*.

† *Data expressed as medians (interquartile range); statistically significant differences between the Normal LBM subgroup and the Low LBM subgroup were determined using the Mann Whitney U-test (p < 0.05)*.

‡ *Echogenicity is expressed via grayscale values (0–255)*.

### Procedures

The primary estimate of LBM was obtained via whole body DXA imaging using a GE Lunar iDXA machine (GE Medical Systems Ultrasound & Primary Care Diagnostics, LLC, Madison, WI, USA). A single trained DXA technician administered all DXA examinations using the GE Encore v15 SP2 software package for the LBM data acquisition and analysis. The body composition data collected during the DXA examinations included estimates of absolute and percentage of total LBM, aLM/ht^2^, and body fat percentage (BF%). The aLM values were calculated as the sum of LBM in the arms and legs and scaled to height (aLM/ht^2^). Participant preparation and positioning for DXA was according to the GE DXA machine manufacturer's manual and the GW Exercise Science Laboratory testing procedures. DXA scans were obtained on the same day as the diagnostic ultrasound examination. Similar DXA imaging equipment and examination procedures (Hull et al., 2009) have yielded reliable measurement results (ICC = 0.97, *p* < 0.0001; CV = 5.5% for LBM) (Aasen et al., 2006).

Sonographic estimates of LBM (aggregate muscle thickness, cm) and myosteatosis (echogenicity levels expressed as grayscale values, 0–255) were obtained by a single trained and certified sonographer. Image capture was completed using a portable, diagnostic ultrasound device (SonoSite M-Turbo 1.1.2; SonoSite, Inc., Bothell, WA, USA) with a 13.6 MHz linear array transducer and B-mode scanning. Ample amounts of water-soluble transmission gel was applied to the transducer in order to maintain adequate acoustic contact with the skin surface. Minimal examiner pressure was exerted during the scanning to attain sufficient image resolution while incurring nominal tissue deformation. The unilateral (Abe et al., 1994) axial and appendicular sites included the midpoint of the upper trapezius, upper pectoralis major, lateral deltoid, proximal forearm (mobile wad compartment), and rectus femoris (dominant side only) as identified via palpation of surface anatomy and confirmed via real-time sonography. Imaging was completed while the participants were seated with their feet on the floor and upper arms relaxed and aligned with the trunk. Their elbows, hips, and knees were positioned with approximately 90° of flexion. These anterior locations were determined by considering accessibility during their future use with non-ambulatory patients, the targeted region of interest (ROI) relative to the ultrasound imaging window and depth, previous use in other investigations, or clear anatomical landmarks that aid the imaging process (Bemben, 2002; Ismail et al., 2014). All longitudinal view images were obtained and measured 3 times using digital calipers within the fascial boarders of the muscle at the time of image capture, and the values were averaged prior to analysis. Acceptable intra-rater reliability (Bemben, 2002; O'sullivan et al., 2007) for diagnostic ultrasound assessment has been found for tests involving the thickness and cross-sectional area of the rectus femoris [ICC_(3, 2)_ = 0.72–0.99, *p* < 0.05; CV = 3.5–6.7%] and similar morphology measures for the trapezius have also been reported as reliable [ICC_(3, 3)_ = 0.88–0.96, *p* < 0.05]. Also, the investigators involved in this study demonstrated a CV of 1.6–2.9% for material thickness measures across 6 raters using a calibration phantom (Harris-Love et al., 2014b) and high interrater reliability [ICC_(2, *k*)_ = 0.992–0.996, *p* < 0.001] for the assessment of echogenicity at the rectus femoris via grayscale histogram analysis (Harris-Love et al., 2015b).

Additional assessments included hand grip dynamometry (Jamar, Lafayette Instruments, Lafayette, IN) using the mean value of 3 trials under standardized conditions (Günther et al., 2008). Grip strength is a frequently used impairment measure in studies concerning general muscle function and older adults (Vermeulen et al., 2015), and the reliability of the Jamar dynamometer is suitable for clinical research settings (ICCs = 0.97–0.98, *p* < 0.01). Basic anthropometric measures such as height (cm) with a stadiometer and body mass (kg) with a balance scale were completed prior to body composition testing, and participants provided general information concerning racial/ethnic group identify, limb dominance (based on the stated preference for handwriting and kicking a ball), past medical history, alcohol intake (The Alcohol Use Disorders Identification Test, AUDIT-C) (Bradley et al., 2007), health-related quality of life (The Health Assessment Questionnaire, HAQ) (Bruce and Fries, 2003), and smoking behavior.

### Data analysis

Descriptive statistics are used to depict participant characteristics and the outcome measures, and data are expressed as means and standard deviations. The major outcomes in this study have normal data and variance distributions based on the Shapiro-Wilk and Levene's test, respectively, except for the ultrasound echogenicity grayscale values and BMI. These data are shown as median values with the interquartile range (IQR) and further analyses are completed using non-parametric statistics or log_10_(*x*) data transformations (Portney and Watkins, 2009). Inferential statistics include an analysis of relationships among the measures of body composition and muscle performance. Pearson product-moment correlation coefficients (PMCC, *r*), partial correlations (*r*_xyz_), and Spearman's correlation coefficients (Spearman's rho, ρ) are used to assess the association between variables, and the strength of the association among the variables is based on Munro's criteria (Munro, 2001). Independent *t*-tests and Mann Whitney *U*-tests are used to determine the difference among the variables based on the categorization of participants in “Normal LBM” and “Low LBM” subgroups. The LBM criterion is based on the Class I designation for sarcopenia in women (5.76–6.75 kg/m^2^) by Janssen and colleagues (Janssen et al., 2004).

Nested linear multiple regression with *a priori* variable selection is used to assess the presumed association of LBM with measures of body size, ultrasound morphometry measures of muscle thickness, and age. Significant improvements in the regression models are based of the change in *F*-values derived from an analysis of variance (ANOVA). Stepwise multiple linear regression analysis is used to determine the association of muscle strength with LBM, echogenicity, body size, body fat (BF), and age. Data residuals are assessed for homoscedasticity and Cook's Distance scores are assessed to ensure that individual data are not disproportionately influencing the regression equation. Multicollinearity of the covariates is initially assessed through the review of a correlation matrix, and then calculating the variance inflation factors (VIF), tolerance statistics (1/VIF), and the covariate dependency associated with each eigenvalue following the regression analysis (Field, 2009). VIF values = 10 denote multicollinearity, and an average VIF > 1 or 1/VIF < 0.1 prompts the review of the variance proportions associated with the eigenvalue dimensions for the final regression model. Covariate dependency observed within any eigenvalue dimension will also serve to confirm the presence of multicollinearity.

The construct of “strength” is represented by the averaged peak grip force values scaled to body weight given the well-known influence of body size on the expression on unadjusted strength values (kg of peak force/kg of body weight) (Jaric, 2002, 2003). Echogenicity measures are expressed as median grayscale values (a unitless 0–255 scale, with higher values indicating more hyperechoic material) via image analysis using Adobe Photoshop® version 6 (Adobe Systems, Mountain View, CA, USA) (Harris-Love et al., 2015b). Total sample data and/or subgroup data were subject to analysis based on the nature of a given research question associated with the study objectives. Statistical analyses were performed using SPSS statistical software version 10.0 for Windows (SPSS Inc., Chicago, IL, USA). The α level was set at 0.05, and two-tailed *p* < 0.05 were considered significant for all inferential statistics.

## Results

### Participant characteristics

Our sample includes 20 female participants with a mean age of 43.4 ± 20.9 years with a median BMI of 23.8 (IQR, 8.5) and a mean aLM/ht^2^ of 6.96 ± 1.22. Ratings of health-related quality of life via the HAQ were similar to those reported in population-based studies, no excessive alcohol intake was detected using the Audit-C questionnaire, and no participant reported a history of smoking (Bruce and Fries, 2003; Bradley et al., 2007). The assignment of participants to Normal LBM and Low LBM subgroups reveals that the Normal LBM subgroup exhibit higher BMI values (*p* = 0.001) and echogenicity levels (*p* = 0.003), but lower scaled grip strength values (*p* = 0.017) in comparison to the Low LBM group. Ultrasound estimates of LBM via aggregate total muscle thickness values significantly discriminate between the Normal LBM and the Low LBM subgroups (*p* = 0.006). All participant characteristics and demographic information are provided in Table 1.

### Using ultrasound muscle characteristics to improve predictors of lean body mass

While ultrasound morphometry measures are independently associated with LBM (*r* = 0.64, *p* = 0.002), a multiple regression model using the aggregate ultrasound muscle thickness measures with estimates of body size and participant age provides the strongest association with DXA LBM values. The iterations of the linear regression model show that BMI alone is a predictor of aLM/ht^2^ [adjusted *R*^2^ of 0.61, *p* < 0.001, using log_10_ (*x*) values for BMI]. However, the model is significantly improved [Δ*R*^2^ = 0.13, *F*_(2, 17)_ = 32.5, *p* < 0.004] with the addition of aggregate ultrasound muscle thickness (adjusted *R*^2^ of 0.77, *p* < 0.001) and age [Δ*R*^2^ = 0.08, *F*_(3, 16)_ = 35.4, *p* < 0.007] as predictor variables. The *a priori* regression model of BMI, ultrasound muscle thickness, and age yields an adjusted *R*^2^ of.85 (*p* < 0.001; Table 2). The partial correlations within this model show the strength of association between BMI and aLM/ht^2^ (*r*_xyz_ = 0.88). Contributing predictor variables, ultrasound muscle thickness and age, exhibit a similar magnitude of association with aLM/ht^2^ (*r*_xyz_ = 0.58 and −0.61, respectively). In examining the potential presence of multicollinearity within the regression model, the 1/VIF was 0.66–0.76 and the VIF was 1.3–1.5. The variance proportions associated with the eigenvalue dimensions do not reveal covariate dependency. The highest regression coefficient variances observed across all eigenvalue dimensions are for BMI (0.97) and age (0.16) within eigenvalue dimension 4 of the final regression model.

**Table 2:** **Regression model for aLM/ht^2^**.

**Model**	***r***	***R*^2^**	**Adjusted *R^2^***	**Std. Error of the Estimate**	***F***	**Sig**.
1	0.81	0.66	0.61	0.731	35.1	<0.001
2	0.89	0.79	0.77	0.588	32.5	<0.001
3	0.93	0.87	0.85	0.482	35.4	<0.001

*The linear regression model features lean body mass obtained from dual energy X-ray absorptiometry (DXA) as the dependent variable and the body mass index (BMI) with the aggregate muscle thickness value (US) and age as predictor variables*.

*This a priori model utilized a nested linear, multiple regression model with forward entry—Predictors: (1) log_10_ (BMI); (2) log_10_ (BMI) + aggregate muscle thickness (via ultrasound, cm); (3) log_10_ (BMI) + aggregate muscle thickness (via ultrasound, cm) + age (years). Dependent Variable: DXA lean body mass (aLM/ht^2^). Model 3: Y = −9.078 + 10.210(log_10_ (BMI)) + 0.302(US) + −0.019(age)*.

### Muscle quality estimates, body composition estimates, and peak force generation

Estimates of muscle quality, proportion of total body fat, and age, but not LBM, are significantly associated with scaled peak force production. Peak force generation was represented by dominant limb grip dynamometry scaled to body weight in our sample (differences between dominant and non-dominant strength values were not significant; data not shown). Participant age and ultrasound echogenicity measured at the dominant limb rectus femoris are moderately associated with strength (*r* = −0.69, *p* = 0.001, and ρ = −0.67, *p* = 0.001, respectively). Considering the body composition measures obtained using DXA, percentage body fat (BF%) is moderately associated with scaled peak force (*r* = −0.63, *p* = 0.003), but LBM as estimated with aLM/ht^2^ is not (*r* = −0.34, *p* = 0.14).

**Figure 1:**
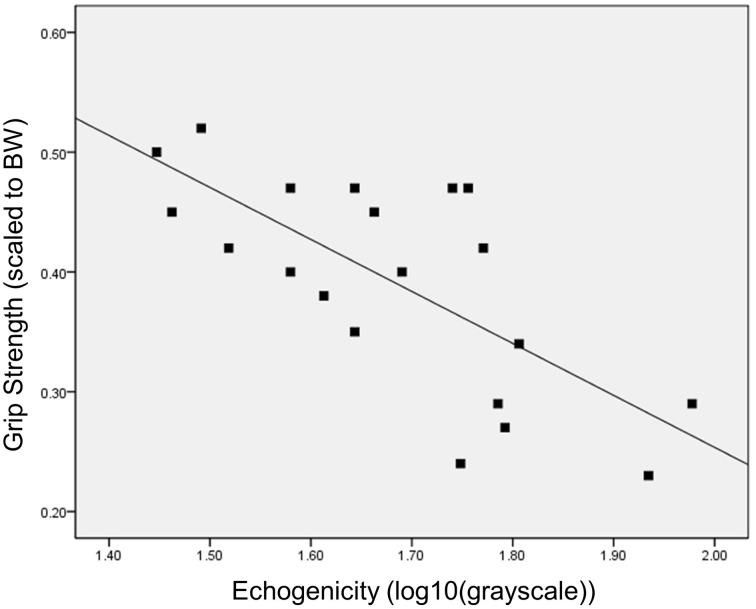
**Bivariate relationship between grip strength and muscle echogenicity**. The scatterplot depicts the inverse relationship between grip strength (peak force scaled to body weight) and muscle quality as measured via grayscale histogram analysis of the rectus femoris echogenicity.

The bivariate linear regression model with age as a predictor of scaled peak force yields an adjusted *R*^2^ of 0.39, *p* = 0.002. The addition of ultrasound echogenicity, as quantified with grayscale histogram analysis [using log_10_ (*x*) grayscale values], significantly improves the model [Δ *R*^2^ = 0.16, *F*_(2, 18)_ = 11.8, *p* = 0.017]. The multiple regression model with age and echogenicity as predictor variables accounts for approximately 53% of the variance in the scaled peak force values (*p* = 0.001; Table 3). The partial correlations within this model suggest that echogenicity may have a greater magnitude of association with scaled peak force (*r*_xyz_ = −0.52) in comparison with participant age (*r*_xyz_ = −0.38). The addition of other predictor variables associated with body size and body composition, such as BMI and BF%, only serve to diminish the integrity of regression model (*F*-value decreases from 13.3 to <7.9 without a resultant increase in the adjusted *R*^2^-value). Regression model diagnostics are negative for multicollinearity based on a 1/VIF of 0.62, a VIF of 1.6, and an absence of covariate dependency within the eigenvalue dimensions. Figure 1 depicts the scatterplot for scaled peak force and echogenicity expressed as grayscale values (log_10_(*x*)).

**Table 3:** **Regression model for grip strength**.

**Model**	***r***	***R^2^***	**Adjusted *R^2^***	**Std. Error of the Estimate**	***F***	**Sig**.
1	0.65	0.42	0.39	0.068	13.26	0.002
2	0.76	0.58	0.53	0.059	11.75	0.001

*The linear regression model features peak force obtained via grip dynamometry and scaled to body weight as the dependent variable and subject age and ultrasound echogenicity as estimated via grayscale analysis as the predictor variables*.

*This model utilized a nested linear multiple regression with forward variable entry—Predictors: (1) age; (2) age + log_10_ (echogenicity via grayscale). Dependent Variable: grip strength (scaled to body weight; dominant side). Model 2: Y = 0.969−0.306(log_10_ (echogenicity using grayscale values)) −0.001(age)*.

## Discussion

Age-related muscle dysfunction may be marked by both a loss of LBM and diminished muscle tissue composition. While the assessment of muscle quality is not yet included in the staging algorithm for sarcopenia (Lee et al., 2013), intrinsic muscle characteristics beyond size are known to affect strength and contribute to mobility limitations (Goodpaster et al., 2001; Ferrucci et al., 2011). Mobile, diagnostic ultrasound has been proposed as a method to obtain estimates of muscle mass and muscle quality, while circumventing the constraints of traditional imaging modalities related to access, cost, and radiation exposure (Pillen and van Alfen, 2011; Harris-Love et al., 2014c). The primary objectives of this study are to examine if diagnostic ultrasound muscle characteristics help to improve well-known determinants of LBM, and if the measurement of muscle quality via ultrasound echogenicity is associated with muscle performance.

### Diagnostic ultrasound and LBM estimates: improving on available clinical information

Standard clinical information such as age and BMI are significantly associated with LBM, but fall short of full consideration as proxy measures. Our data is consistent with the findings of a larger study conducted by Iannuzzi-Sucich and colleagues (Iannuzzi-Sucich et al., 2002) who determined that BMI independently accounts for approximately 50% of the variance in aLM/ht^2^. Also, Goodman and associates (Goodman et al., 2013) have used logistic regression models with factors for BMI and age to identify older men and women with low aLM/ht^2^ based on data culled from the National Health and Nutrition Examination Surveys database (1999–2004) and comparisons with a young cohort reference group. In this study, we have used a conceptual aLM/ht^2^ prediction model based on BMI, age, and a direct measure of muscle morphometry via diagnostic ultrasound. The general use of BMI remains problematic (Romero-Corral et al., 2008; Harris-Love et al., 2014a) concerning the misclassification of very fit individuals as “overweight,” its potential overestimate of obesity rates in African Americans, and the wide range of BF% levels attributed to people with a BMI range between 20 and 30. However, the value of retaining BMI within the proposed aLM/ht^2^ prediction model is its significant association with LBM in many patient populations, and its representation of body size which serves to provide a scaling factor for the aggregate muscle thickness values obtained via sonography. An additional potential benefit of using diagnostic ultrasound data for an aLM/ht^2^ prediction model, and during the general sarcopenia assessment process, is the viable opportunity to integrate estimates muscle quality into the sarcopenia staging algorithm. The development of valid predictive models of LBM still remains an important goal concerning the staging of sarcopenia and the monitoring of other chronic conditions. Indeed, low LBM and muscle performance constitute health concerns that may act as independent mortality risk factors (Alexandre et al., 2014). Nevertheless, muscle quality may surpass muscle mass as a contributor to age-related decreases in muscle strength and power, and negatively impact functional independence (Fukumoto et al., 2012; Watanabe et al., 2013; Rech et al., 2014). Additional investigation will be needed to refine the operational definitions of muscle quality and to understand how to best incorporate this muscle characteristic into the sarcopenia syndrome framework.

### Muscle quality should not be ignored as a component of the sarcopenia syndrome

**Figure 2:**
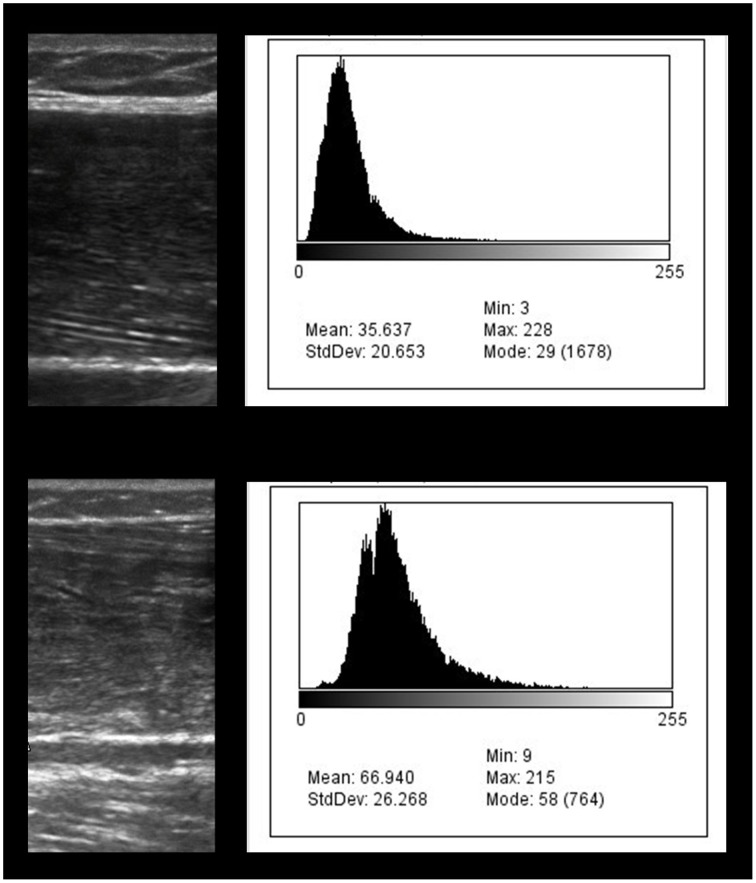
**Diagnostic ultrasound image of the rectus femoris region of interest and the corresponding grayscale histogram analysis values**. The exemplar images depict the diagnostic ultrasound transverse muscle images on the left and the grayscale histograms on the right. The bottom ultrasound image shows greater hyperechoic properties in comparison to the top image. The comparatively hyperechoic image characteristics of the bottom image correspond to grayscale histogram data with a wider distribution and a shift to the right which is associated with larger grayscale values. The grayscale value of the bottom image is 66.9 and may indicate a greater proportion of intramuscular adipose tissue in comparison to the top image (grayscale value, 35.6).

Older adults categorized as mildly overweight based on their BMI are less likely to develop sarcopenia using LBM as the criterion (Yu et al., 2014). Individuals that are mildly overweight may exhibit a protective effect against muscle loss and maintain functional independence as they age despite a concomitant increased risk for cardiovascular disease and other systemic disorders (Cetin and Nasr, 2014). Indeed, BMI significantly (*p* = 0.001) discriminates between participants in this study assigned to the Normal LBM subgroup (>6.75 kg/m^2^) and Low LBM subgroup (5.76–6.75 kg/m^2^). The Normal LBM subgroup has a mean LBM value of 7.92 ± 0.88 kg/m^2^ and a BMI of 28.8 (IQR, 9.4), whereas the Low LBM subgroup has a mean LBM value of 6.00 ± 0.55 kg/m^2^ and a BMI of 21.5 (IQR, 3.1). Therefore, the Normal LBM subgroup appears to reflect previously published findings concerning the LBM sparing effect of higher relative body weight levels. Nevertheless, the Normal LBM subgroup also exhibits *lower* scaled peak force values and *higher* echogenicity values in comparison to the Low LBM subgroup (Figure 2). The women assigned to the Low LBM subgroup are classified as having “healthy body weight” per the BMI designation, and they also have a lower proportion of total body fat, higher relative strength levels based on grip dynamometry, and better estimates of muscle quality (i.e., 35% lower echogenicity levels in comparison to the Normal LBM subgroup; Table 1).

While forms of muscle quality are not part of the current sarcopenia staging algorithm, the concept remains useful for examining contributing factors to muscle performance. Muscle quality in sarcopenia studies is sometimes expressed as peak force generated from a single testing maneuver scaled to regional DXA estimates of muscle mass (Cawthon et al., 2009; Hairi et al., 2010). Scaling net muscle force production relative to muscle mass or body mass allows one to compare strength within a heterogeneous sample regarding body stature, and account for the effect of body size on strength-function relationships (Jaric, 2003). Recently, the investigators involved in the Foundation for the National Institutes of Health (FNIH) Sarcopenia Project examined grip strength cut points related to mobility limitations. Although they opted to affirm the use of absolute strength values in a manner similar to other international sarcopenia consensus groups (Lee et al., 2013), they did note the modest improvements in the model equations for women within their pooled cross-sectional sample when using grip strength scaled to BMI (Alley et al., 2014). While, the aforementioned scaling approach has been termed “specific force” in previous studies (Cawthon et al., 2009; Hairi et al., 2010), there may be important distinctions between scaling factors and specific force that merit consideration. Specific force has traditionally been determined by calculating muscle strength relative to whole muscle cross-sectional area (CSA), and is usually depicted as a simple linear relationship that may have some validity in unipennate muscles with fairly uniform architecture. However, the assumptions of specific force derived from CSA estimates do not apply to the vast majority of muscle groups. Consequently, specific force is often formally expressed as the quotient of muscle force and physiologic cross-sectional area (PCSA), which incorporates aspects of muscle architecture such as muscle fiber length and pennation angle (Blemker et al., 2005; Morse et al., 2008; Krivickas et al., 2011). Additional intrinsic factors such as moment arm length, muscle fiber type, muscle action mode, bioenergetics, excitation-contraction coupling, and muscle tissue composition act to influence specific force. Furthermore, factors extrinsic to the muscle—but inextricably linked with net force production—include sufficient cortical excitability, the integrity of pyramidal neurons, the synchrony and rate coding of alpha motor neurons, and the impact of age-related motor neuron loss (Manini et al., 2013; Lieber, 2010). Given the varied physiological factors that govern muscle performance, these insights imply that the use of specific force to represent muscle quality has important constraints. Rather, the calculation of specific force could be considered as one of many impairment-level outcomes that are responsive to changes in muscle quality and other facets of the neuromuscular milieu.

In this report, muscle quality is operationally defined as muscle tissue echogenicity which serves as a proxy measure for tissue composition (Reimers et al., 1993; Sipilä and Suominen, 1993). The rationale for considering diminished tissue composition as a major indicator of age-related muscle changes is partially validated through the significant inverse relationship between scaled peak force and echogenicity observed in our data (Figure 1). Given that LBM did not have a meaningful association with scaled peak force, and that age and echogenicity accounted for approximately 50% of the variance in strength levels, our pilot data allows for the consideration of additional intrinsic and extrinsic muscle factors contributing to the observed strength levels within the sample.

### Study implications and limitations

The findings from this study suggest that diagnostic ultrasound may be used in combination with readily available clinical information to estimate LBM. Although the models derived from the data must be considered exploratory given the limited sample size, the *a priori* explanatory variables lend strength to our general approach (Field, 2009). While the coefficients used in the regression equations may change substantially during validation with a larger sample and with the inclusion of male subjects, we hypothesize that the explanatory variables of BMI, ultrasound muscle thickness, and age will retain their value within the model. Use of the Class I designation for sarcopenia in women (i.e., 5.76–6.75 kg/m^2^) is appropriate for our participants given their relatively high level of physical functioning, and serves as an approach to discriminate meaningful body composition differences within the sample (Janssen et al., 2004). More stringent LBM criterion values, such as those ascribed to the Class II sarcopenia designation or the FNIH sarcopenia staging algorithm, yield lower prevalence values (Clynes et al., 2015) and may be more suitable for population-based studies with a sufficient representation of participants with a high degree of physical impairment.

Muscle echogenicity was significantly associated with peak muscle force in our sample. It is important to note that the sonographic morphology measures used for the proxy muscle tissue composition estimates were obtained at the rectus femoris. The selection of the rectus femoris for echogenicity assessment is influenced by its favorable architecture and uniform geometry in the longitudinal orientation during scanning. Previous observations confirm that echogenicity of skeletal muscles vary with their location within the body, with muscle groups within the lower compartment of the leg having higher echogenicity in comparison to selected upper body muscle groups (Scholten et al., 2003; Pillen and van Alfen, 2011). We hypothesized that while skeletal muscles have differing levels of echogenicity based on their location and metabolic profile, age-related changes in muscle tissue composition would be systemic and result in a broad increase in echogenicity across muscle groups. This proposed phenomenon is partially supported by our findings in this study concerning the observed significant relationship between echogenicity at the rectus femoris with peak grip force. Just as grip strength has been used as a global measure that may be significantly associated with knee extension strength and general physical performance in older adults (Aadahl et al., 2011; Cooper et al., 2013), echogenicity at the knee extensors may be a general indicator of muscle quality that is inversely related with grip strength and general measures of muscle performance. For example, our preliminary data (Harris-Love et al., 2015a) involving a group of older men suggest that echogenicity levels at the rectus femoris are significantly related to scaled peak grip strength, walking speed, and the timed sit-to-stand test (*r* = −0.30 to −0.71, *p* < 0.05). Further study will be needed to better understand the effect of sexual dimorphism on the age-related changes in muscle tissue composition as assessed with sonographic proxy measures. Also, larger follow up studies will be needed to explore the risk of incident mobility limitations and physical disability based on muscle quality estimates as described in this work.

Investigators have also reported findings that suggest that changes in muscle tissue composition may differentially affect people of African descent (Song et al., 2004; Miljkovic-Gacic et al., 2008; Miljkovic et al., 2009). Both advancing age and BF% may be associated with adverse changes in muscle tissue composition. However, high levels of intramuscular adipose tissue in African Americans may be observed in those classified as having “healthy body weight” based on their BMI, and be independent of central adiposity (Miljkovic-Gacic et al., 2008). Individuals with this type of muscle tissue composition profile may have associated health problems that include metabolic dysfunction or diminished muscle performance, and yet not meet the staging criteria for sarcopenia. Indeed, there is some evidence to suggest that African Americans may have a lower prevalence of sarcopenia in comparison to non-Hispanic Whites (Castaneda and Janssen, 2005). We do not have a sufficient sample size to subject our racial/ethnic group data to inferential analysis. However, we observed that none of our African American or Hispanic participants are in the Low LBM subgroup (Table 1). These 6 participants are in the Normal LBM subgroup which is characterized by higher mean BMI and median echogenicity values in comparison to the Low LBM subgroup. Other limitations in this work related to the modest sample size include the departures from normality related to the distribution of the BMI and grayscale values which was addressed via data transformation. Also, the constraints of standard diagnostic ultrasound imaging did not allow for us to obtain the additional measures of CSA or PSCA at the mid-thigh. While grip dynamometry is the recommended means of strength testing according to the leading sarcopenia consensus organizations (Cruz-Jentoft et al., 2010; Cruz-Jentoft and Morley, 2012; Dam et al., 2014), the study findings may have been enhanced by obtaining estimates of lower extremity muscle performance.

It remains to be seen if screening for age-related changes in muscle quality may be effectively used to modify the risk of developing chronic disease and disabling conditions related to musculoskeletal health. In addition, the benefits of diagnostic ultrasound to characterize skeletal muscle have to be considered with the shortcomings of the imaging modality related to equipment access, examiner training, limited normative datasets, and the inter-machine equivalence of echogenicity values (Harris-Love et al., 2014c).

## Conclusions

Diagnostic ultrasound may provide a clinically viable means to assess both muscle mass and muscle quality. Our study findings indicate that a conceptual aLM/ht^2^ prediction model based on BMI, age, and a direct measure of muscle morphometry via diagnostic ultrasound, accounts for 85% of the variance in DXA LBM values for our sample. Moreover, our data suggest that age and muscle echogenicity, are significantly associated with scaled peak force production in the women that participated in our study. In contrast, DXA LBM is not significantly associated with scaled peak force generation in our participants. The higher total BF% of the Normal LBM subgroup may have conferred a protective effect against low muscle mass, but not myosteatosis. The women in the Normal LBM subgroup exhibit higher BMI values and echogenicity levels, but lower scaled peak force values in comparison to the Low LBM group. Follow up studies should include validation of the aLM/ht^2^ prediction model, and the integration of ultrasound estimates of muscle quality into the sarcopenia staging algorithm.

## Author contributions

MH and CI were responsible for the study design; CI, JZ, PW, HM and MH performed the study procedures; CI, CT, HH, and MH were responsible for data management and verification; MH analyzed the data and prepared figures; MH, CI, LD, and MB collaborated on the data interpretation; MH and CI drafted the manuscript; CI, JZ, HH, PW, HM, CT, LD, MB, and MH edited and revised the manuscript; CI, JZ, HH, PW, HM, CT, LD, MB, and MH approved final draft.

### Conflict of interest statement

The authors declare that the research was conducted in the absence of any commercial or financial relationships that could be construed as a potential conflict of interest.
